# Changes in risk behaviour following a network peer education intervention for HIV prevention among male Tajik migrants who inject drugs in Moscow: a cluster‐randomized controlled trial

**DOI:** 10.1002/jia2.26310

**Published:** 2024-07-19

**Authors:** Mary Ellen Mackesy‐Amiti, Mahbatsho Bahromov, Judith A. Levy, Jonbek Jonbekov, Casey M. Luc

**Affiliations:** ^1^ School of Public Health University of Illinois Chicago Chicago Illinois USA; ^2^ PRISMA Research Center Dushanbe Tajikistan

**Keywords:** HIV prevention, peer education intervention, cluster‐randomized trial, injection drug use, Tajik, migrant worker

## Abstract

**Introduction:**

The “Migrants’ Approached Self‐Learning Intervention in HIV/AIDS for Tajiks” (MASLIHAT) recruits and trains Tajik labour migrants who inject drugs as peer educators (PEs) in delivering HIV prevention information and encouragement to adopt risk‐reduction norms and practices within their diaspora social networks while reducing their own HIV risk.

**Methods:**

The MASLIHAT intervention was tested in Moscow in a cluster‐randomized controlled trial with 12 recruitment sites assigned to either the MASLIHAT intervention or an equal‐time peer‐educator training focused on other health conditions (TANSIHAT). From October 2021 to April 2022, 140 male Tajik migrants who inject drugs were recruited as PEs to attend the 5‐session MASLIHAT training or the TANSIHAT non‐HIV comparison condition. Each participant in both groups recruited two network members (NMs) who inject drugs with the intent to share with them the information and positive strategies for change they had learned (*n* = 280). All PEs and NMs (*n* = 420) participated in baseline and follow‐up interviews at 3‐month intervals for 1 year. All received HIV counselling and testing. Modified mixed effects Poisson regressions tested for group differences in injection practices, sexual risk behaviours and heavy alcohol use over time.

**Results:**

At baseline, across both groups, 75% of participants reported receptive syringe sharing (RSS), 42% reported condomless sex and 20% reported binge drinking at least once a month. In contrast to TANSIHAT where HIV risk behaviours remained the same, significant intervention effects that were sustained over the 12 months were observed for receptive syringe and ancillary equipment sharing among both MASLIHAT PEs and NMs (*p* < 0.0001). Significant declines in the prevalence of sexual risk behaviours were also associated with the MASLIHAT intervention (*p* < 0.01), but not the comparison condition. Binge alcohol use was not affected in either condition; the MASLIHAT intervention had a transitory effect on drinking frequency that dissipated after 9 months.

**Conclusions:**

The MASLIHAT peer‐education intervention proved highly effective in reducing HIV‐related injection risk behaviour, and moderately effective in reducing sexual risk behaviour among both PEs and NMs. Network‐based peer education is an important tool for HIV prevention among people who inject drugs, especially in environments that are not amenable to community‐based harm reduction.

## 1 INTRODUCTION

While significant progress has been made in addressing the AIDS pandemic throughout much of the world, new cases of HIV increased by 48% in eastern Europe and central Asia from 2010 to 2021 [1], with most new cases occurring in the Russian Federation [2]. Although the HIV epidemic in the Russian Federation has become generalized [3], injection drug use still accounts for about 40% of new cases [1, 4]. Labour migrants who inject drugs while in Russia are at especially high risk for acquiring HIV due to social marginalization and lack of access to healthcare and prevention services [5]. Many migrant workers in Russia originate from the central Asian countries, including Tajikistan—a small country with comparatively lower HIV rates, high poverty and an ongoing opioid epidemic [6, 7, 8].

We developed the Migrants’ Approached Self‐Learning Intervention in HIV/AIDS for Tajiks (MASLIHAT) intervention to address the need for preventive interventions for this population [9]. MASLIHAT is a network‐based, peer educator (PE) training intervention developed as a socio‐cultural adaptation of the Self‐Help in Eliminating Life‐Threatening Diseases (SHIELD) model [10, 11, 12] to reduce risky drug and sexual behaviour among male Tajik migrants in Moscow who inject drugs. Heavy alcohol use that may contribute to sexual risk through disinhibition is also targeted. Like SHIELD, MASLIHAT is designed to promote the dissemination of information and behavioural risk‐reduction modelling through social networks to produce changes in social norms of HIV‐related sexual and drug injection risk behaviour. It also draws on Yang's Theory of Migration [13] that emphasizes the need to modify the psychosocial conditions and life circumstances that contribute to risk behaviour. Presently, the intervention targets only male migrants, as the number of female Tajik migrants who inject drugs is quite small, and Tajiks would be uncomfortable discussing sexual risk in a mixed‐sex group. Pilot testing in 2018 demonstrated promising results with significant declines in HIV risk behaviour over 6 months among both PE participants and their network members (NMs) with whom they regularly interacted [9].

In the present study, a cluster‐randomized parallel groups trial tests the efficacy of the MASLIHAT intervention versus a comparison condition in reducing HIV‐related risk behaviour. We adapted an existing health education intervention (Healthy Living) previously developed to serve as a control intervention to create the “Targeted Application of Network and Social Intervention on Health Assistance for Tajiks” (TANSIHAT) as an equal‐time health education intervention focusing on other relevant health conditions such as tuberculosis (TB) and cardiovascular disease but not HIV. In both conditions, participants were trained as PEs and referred for HIV counselling and testing at the Moscow HIV Prevention Center [14]. Risk behaviour was assessed at baseline and at 3‐month intervals during 1 year of follow‐up.

## 2 METHODS

Study procedures were reviewed and approved by the Institutional Review Boards of the University of Illinois Chicago, PRISMA Research Center in Tajikistan, and the Moscow Nongovernment Organization “Bridge to the future.” All participants provided written informed consent. All activities and assessments were conducted in Tajik or Russian by male Tajik staff. English‐language instruments were translated by PRISMA investigators/staff and independently back‐translated to English for verification. PRISMA staff are themselves former Tajik migrant workers and are trained on the importance of treating people who inject drugs (PWID) with dignity and compassion.

### 2.1 Recruitment and site assignment

From October 2021 to April 2022, 140 male Tajik migrant workers were recruited and trained as PEs from 12 sites in Moscow: two Tajik diaspora organizations, four bazaars and six construction work sites. To participate as a PE assigned to either the MASLIHAT intervention or the TANSIHAT comparison condition, prospective participants needed to be a male Tajik migrant aged 18 or older, a current or former PWID, give informed consent, intend to reside in Moscow for the next 12 months to participate in their assigned intervention and follow‐up data collection, and willing to recruit two male PWID to participate as NMs for baseline and follow‐up interviewing but who would not participate in either condition's educational sessions or activities. NMs (*n* = 280) had to meet the same eligibility criteria as PEs but also: (1) have injected drugs at least once in the last 30 days; and (2) be someone whom the PE sees at least once a week to permit him to share intervention information and encourage normative and behavioural change within their social networks. Participants received the equivalent of $20.00 in Russian Rubles for their time and transportation costs in participating in intervention sessions (PEs only) and for being interviewed at baseline and follow‐up (both PEs and NMs).

To prevent MASLIHAT cross‐contamination of the control condition through shared peer networks, the 12 recruitment sites were pair‐matched according to site characteristics and randomly assigned to the MASLIHAT versus TANSIHAT condition. Recruiters were blinded as to each site's assignment condition. Site assignment was revealed to the local project coordinator only when it was needed for scheduling intervention sessions.

### 2.2 Sample determination

We estimated power in multilevel analyses using PASS 2019 software (v19.0.1) based on effects observed in the pilot study [9] and assuming up to 10% attrition among intervention participants and up to 15% attrition among NMs. With 12 recruitment sites, intra‐cluster correlation (ICC) = .05, alpha = .01 and at least 10 intervention participants per cluster (20 NMs, 30 total), we estimated at least 80% power to detect medium changes in condomless sex, and over 90% power to detect large changes in syringe sharing. For days of alcohol use, with ICC = 0.3, we estimated at least 80% power to detect a standardized mean difference (SMD) = 0.70 for intervention participants (clusters = 10) and SMD = 0.50 for NMs (clusters = 20).

### 2.3 Intervention sessions

MASLIHAT is a manualized small‐group, interactive intervention that relies on peer networks to reduce drug, alcohol and sexual risk behaviours among temporary migrant workers who inject drugs. Migrants in the host country who inject or previously injected drugs are trained as PEs to promote positive HIV risk‐reduction norms and behavioural change through role modelling and by sharing what they learned during MASLIHAT training sessions with their at‐risk NMs in conversations. The intervention includes five HIV knowledge and skill‐building sessions that involve goal setting, role playing, demonstrations, homework and group discussions. These sessions teach participants techniques for personal HIV risk reduction and the communication and outreach skills needed to encourage others at risk for HIV to also adopt them. MASLIHAT sessions also address general lifestyle, health and safety issues relevant to migrant life [15, 16, 17, 18, 19, 20, 21, 22].

The five sessions are: (1) Introduction to MASLIHAT; general risks and safety for Tajik migrant workers; living a healthy lifestyle, resources & organizations serving Tajik migrants; (2) HIV 101; peer communication skills; (3) HIV/STI risk/prevention through hazardous alcohol consumption/unsafe sex; (4) HIV risk/prevention related to drug use; (5) Maintaining a healthier lifestyle; graduation. Homework and case studies help to script PE messages.

The TANSIHAT programme echoes MASLIHAT in style and time commitment over five sessions: (1) Introduction to TANSIHAT; general risks to health and safety; strategies for general risk‐reduction and living a healthy lifestyle; available resources; (2) Healthy nutrition and personal hygiene; peer communication skills; (3) Fitness and stress management; promoting a healthy lifestyle through physical exercises and stress management; (4) TB risk and prevention among migrants and preventing transmission to families back home; (5) Maintaining healthier living and risk reduction; graduation.

The intervention sessions for both conditions were delivered in groups of 4−7 at the PRISMA Research Center by experienced group facilitators. MASLIHAT and TANSIHAT sessions were delivered by different facilitators. Sessions were scheduled weekly and each lasted 2 hours. Facilitators recorded attendance and rated participant engagement as “not engaged,” “somewhat engaged” or “highly engaged.” Every session started with a homework check‐in. Should a participant miss a session, he received all the session materials and could meet with a session facilitator at a mutually convenient time to ask questions and obtain more information about the missed session. Facilitator presentations and success in stimulating group discussion were observed by PRISMA senior staff and rated on seven facilitator performance dimensions as being poor (0), adequate (1) or good (2).

### 2.4 Baseline and follow‐up interviews

After giving informed consent, baseline interviews with PEs and NMs were conducted at the PRISMA office in Moscow or a private location of the participant's choosing. Comprehensive locator information was collected from participants to aid with follow‐up, including alternate contact information. Following the interview, participants were referred to the Moscow HIV Prevention Center to be tested for HIV and hepatitis C virus (HCV). Anonymized test results were reported to study staff with only a group number to identify the recruitment site. Follow‐up interviews were conducted with PEs and NMs at 3‐month intervals. All participants were referred for repeat HCV testing following the 6‐ and 12‐month interviews and for HIV testing following the 12‐month interview.

### 2.5 Measures

The structured baseline questionnaire collected information on socio‐demographic characteristics, migration characteristics, alcohol use, injection drug use prior to migration and in the past 6 months in Moscow, sexual risk behaviour, and PWID network and injection risk behaviour.

*HIV testing and Serostatus* was assessed at baseline and at each follow‐up by asking: (a) “Have you ever been tested for HIV?” (Yes/No), and if yes, (b) “What were the results of your most recent HIV test?” (1) HIV Positive (you have HIV), (2) HIV Negative (you don't have HIV) and (3) Decline to answer.

*Alcohol use*. Binge drinking was assessed with the question from the Alcohol Use Disorders Identification Test (AUDIT) [23, 24], “How often do you have 6 or more drinks on one occasion,” with responses on a 5‐point scale from “never” to “daily or nearly daily.” Responses were dichotomized for analysis as “never or less than monthly” versus “at least monthly.” Frequency of alcohol use was measured with the question, “How many days in the past month have you used alcohol, including beer, wine, or vodka?”

*Injection risk behaviour*. Recent syringe sharing was assessed in response to the question, “In the past 3 months, how often have you used a needle to shoot drugs after someone else used it first?” with response options: never, rarely, less than half the time, about half the time, more than half the time, almost always and always. Responses were dichotomized into a binary measure of having or not having used a shared syringe within the past 3 months.

*Sexual risk behaviour*. Measures of sexual risk behaviour included condomless sex in the past 3 months, multiple female sex partners and sex with female sex workers (FSWs). Participants were asked for the number of women with whom they had sexual intercourse in the past 30 days, and how many of these were sex workers. Responses were used to create binary measures of multiple female partners and any FSW partner in the past 30 days. Condom use was assessed by asking participants, “how often did you use a condom when having sexual intercourse?” for each of three partner categories: regular female sex partner in Russia, FSW, and other sexual partners not engaged in selling sex. Response categories were “never,” “sometimes,” “often” or “always.” Responses were combined into a binary measure of condomless versus no condomless sex in the past 3 months.

### 2.6 Analysis

We tested the effects of the MASLIHAT intervention on the study's primary outcomes of receptive syringe sharing (RSS), condomless sex and binge drinking. We also analysed additional outcomes of ancillary equipment sharing, multiple female sex partners, sex with sex workers and frequency of alcohol use. Mixed effects modified Poisson regression models with random intercepts for participant and network cluster were estimated for each outcome [27, 28]. The modified Poisson model has the advantage of readily providing covariate‐adjusted risk ratios and standard errors. Time was included as four dummy variables for 3‐, 6‐, 9‐ and 12‐month follow‐up. We tested a three‐way interaction of condition, time and participant type (PE or NM), and non‐significant interactions (*p* > .10) were removed from the model. Marginal contrasts tested for intervention effects by participant type. Unadjusted prevalence ratios with 95% confidence intervals were examined for each outcome. The effects of adjusting for covariates identified as having significant associations at baseline were investigated including number of trips to Moscow, time in Moscow on the current trip, age, and level of education.

## 3 RESULTS

**Figure 1:**
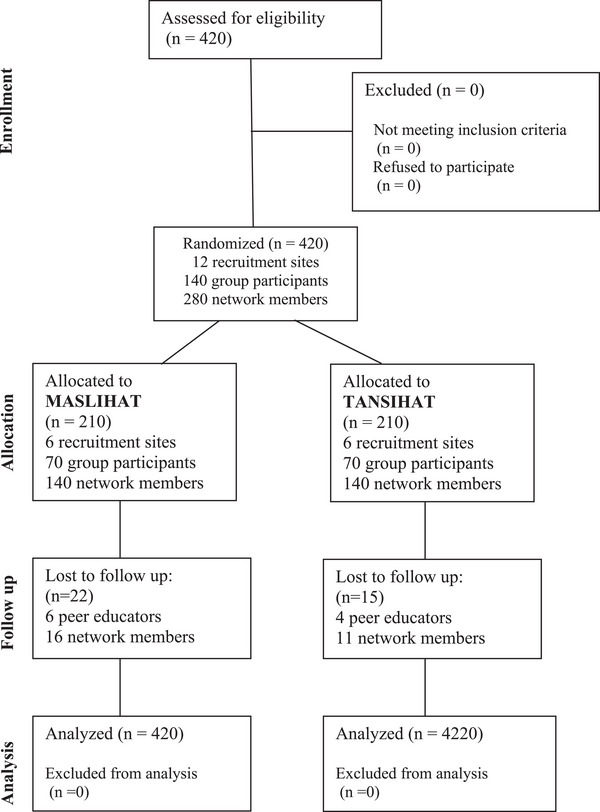
**CONSORT diagram of MASLIHAT cluster‐randomized trial**. Abbreviations: MASLIHAT, Migrants' Approached Self‐Learning Intervention in HIV/AIDS for Tajiks; TANSIHAT, Targeted Application of Network and Social Intervention on Health Assistance for Tajiks (comparison condition).

Table 1 shows the baseline demographic characteristics of PEs (*N* = 140) and NMs (*N* = 280) for the entire sample. There were no significant differences between intervention arms, except on marital status (8.6% currently married in MASLIHAT, 16.2% in TANSIHAT; Chi2 = 8.53, *p* = 0.014). The CONSORT diagram in Figure 1 depicts the two groups’ progress through the multiple phases of the study's parallel randomized trial. The monthly average of drinking days was 5.3 (SD 3.27, Range: 0−20), and 21% reported binge drinking (six or more drinks at a time) at least once a month. Sexual risk behaviour was common with 42% reporting condomless sex in the past month. Over 75% reported injecting with a previously used syringe in the past 3 months. At baseline, 17% reported they had been tested for HIV, and one participant disclosed being HIV positive; 20% of those tested (*n* = 14) declined to disclose their results. Of the 413 participants who were tested for the study, 28 (6.8%) tested HIV positive. All participants were offered help in obtaining HIV treatment. There were no new HIV acquisitions among participants who were tested during follow‐up.

**Table 1:** Demographic characteristics of PWID enrolled in the MASLIHAT trial

	Peer educators (*n* = 140)		Network members (*n* = 280)	
Variable	*Mean (SD)*	Range	*Mean (SD)*	Range
Age	30.7	21−50	29.6	19−49
	(6.74)		(5.90)	
	*n*	%	*n*	%
Recruitment site				
Diaspora organization	24	17.1	48	17.1
Bazaar	44	31.4	88	31.4
Construction site	72	51.4	144	51.4
Area of origin				
Dushanbe	31	22.1	57	20.4
Khatlon	29	20.7	59	21.1
Sughd	13	9.3	31	11.1
Gorno‐Badakhshan	54	38.6	105	37.5
Subordinate districts	13	9.3	28	10.0
Education				
Secondary or less	91	65.0	165	58.9
College or technical college	32	22.9	73	26.1
University but no degree	6	4.3	8	2.9
University degree	11	7.9	34	12.1
Marital status				
Not married	65	46.4	111	39.6
Married	14	10.0	38	13.6
Divorced	61	43.6	129	46.1
Missing			2	0.7
How long in Russia this trip				
One year or less	9	6.4	30	10.7
>1 to 2 years	42	30.0	88	31.4
>2 years	86	61.4	156	55.7
Missing	3	2.1	6	2.1
How many trips to Moscow				
One	8	5.7	41	14.6
Two	55	39.3	81	28.9
Three or more	77	55.0	158	56.4
Employment				
Construction	76	54.3	154	55.0
Loading in bazaar	29	20.7	58	20.7
Selling/food service	27	19.3	48	17.1
Other/Missing	8	5.7	20	7.1

Abbreviations: MASLIHAT, “Migrants’ Approached Self‐Learning Intervention in HIV/AIDS for Tajiks”; PWID, people who inject drugs; SD, standard deviation.

### 3.1 Intervention implementation and attendance

All participants attended at least four of the five sessions, and 81% attended all five sessions. Attendance and facilitator ratings of participant engagement were similar across treatment arms. Facilitators received “good” ratings across all dimensions in 98% of sessions with no difference between treatment arms.

### 3.2 Follow‐up and retention

Over 90% of participants completed all interview waves. Thirty‐seven participants (8.8%) were lost to follow‐up at 9 (*n* = 18) or 12 months (*n* = 19). Loss to follow‐up was similar across treatment arms and participant type.

### 3.3 Risk behaviour outcomes

**Figure 2:**
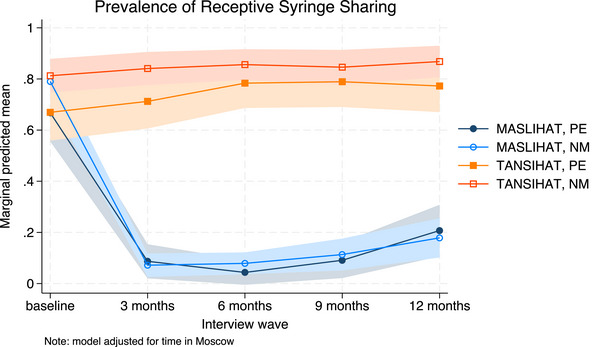
**Predicted prevalence of receptive syringe sharing. Marginal predictions of receptive syringe sharing with 95% confidence intervals, by time, condition and participant type, adjusted for time in Moscow**. Abbreviations: MASLIHAT, intervention condition; NM, network members; PE, peer educators; TANSIHAT, control condition.

**Figure 3:**
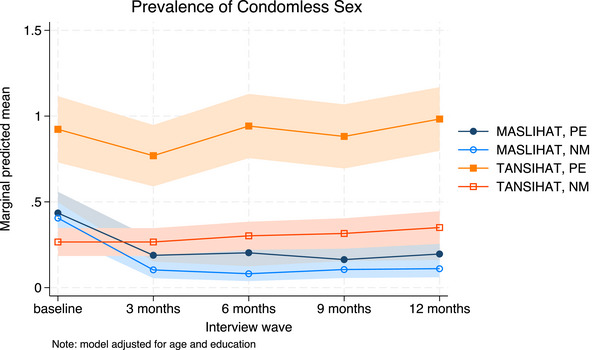
**Predicted prevalence of condomless sex. Marginal predictions of any condomless sex with 95% confidence intervals, by time, condition and participant type, adjusted for age and level of education**. Abbreviations: MASLIHAT, intervention condition; NM, network members; PE, peer educators; TANSIHAT, control condition.

**Figure 4:**
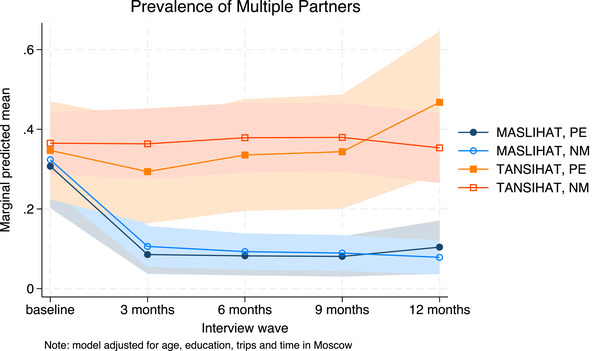
**Predicted prevalence of multiple partners. Marginal predictions of multiple female partners with 95% confidence intervals, by time, condition and participant type, adjusted for trips to Moscow, time in Moscow, age and level of education**. Abbreviations: MASLIHAT, intervention condition; NM, network members; PE, peer educators; TANSIHAT, control condition.

**Figure 5:**
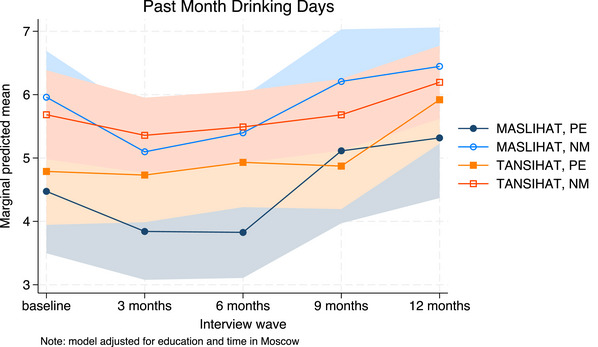
**Predicted drinking days. Marginal predictions of days drinking alcohol in the past month with 95% confidence intervals, by time, condition and participant type, adjusted for time in Moscow and level of education**. Abbreviations: MASLIHAT, intervention condition; NM, network members; PE, peer educators; TANSIHAT, control condition.

Results of the unadjusted Poisson models are shown in Tables 2, 3, 4. We used MASLIHAT and PE as the reference groups so that the time effect shows the difference between follow‐up and baseline for PEs in the MASLIHAT condition. Covariate‐adjusted model results are available in File S1 and marginal predictions are presented graphically in Figures 2, 3, 4, 5.

**Table 2:** Intervention effects on injection risk behaviours, unadjusted mixed effects robust Poisson regression

	Syringe sharing			Equipment sharing		
	*IRR*	*95% CI*	*p‐value*	*IRR*	*95% CI*	*p‐value*
Time^a^						
3 Months	0.13	0.06, 0.28	<0.001	0.11	0.07, 0.17	<0.001
6 Months	0.09	0.03, 0.22	<0.001	0.11	0.07, 0.18	<0.001
9 Months	0.16	0.08, 0.31	<0.001	0.11	0.06, 0.19	<0.001
12 Months	0.33	0.21, 0.51	<0.001	0.12	0.07, 0.19	<0.001
Arm^b^						
TANSIHAT versus MASLIHAT	1.00	0.79, 1.26	1	0.99	0.82, 1.20	0.919
Participant type^c^						
Network member versus PE	1.18	1.00, 1.39	0.045	1.09	0.97, 1.21	0.156
Arm × Participant type^d^	1.03	0.81, 1.29	0.821	−		
Time × Arm^e^						
3 Months	8.33	3.85, 18.02	<0.001	10.55	6.69, 16.65	<0.001
6 Months	13.75	5.22, 36.22	<0.001	10.73	6.31, 18.24	<0.001
9 Months	7.57	3.71, 15.43	<0.001	10.88	6.18, 19.17	<0.001
12 Months	3.53	2.21, 5.64	<0.001	10.64	6.44, 17.68	<0.001
Time × Participant type^f^						
3 Months	0.71	0.32, 1.56	0.390	−		
6 Months	1.16	0.43, 3.14	0.763			
9 Months	0.91	0.49, 1.69	0.774			
12 Months	0.69	0.46, 1.02	0.062			
Time × Arm × Participant type^g^						
3 Months	1.38	0.62, 3.06	0.432	−		
6 Months	0.77	0.29, 2.09	0.612			
9 Months	0.96	0.52, 1.80	0.911			
12 Months	1.35	0.89, 2.04	0.154			
Random intercept variances	*var*	*SE*		*var*	*SE*	
cluster	0	0		0	0	
subject	0	0		0	0	
*N*	420			420		
clusters	140			140		
observations	2039			1973		

Abbreviations: CI, confidence interval; IRR, incidence rate ratio; MASLIHAT, “Migrants’ Approached Self‐Learning Intervention in HIV/AIDS for Tajiks”; NM, network member; PE, peer educator; SE, standard error; TANSIHAT, Targeted Application of Network and Social Intervention on Health Assistance for Tajiks (comparison condition); var, variance.

^a^ Effect of time for PEs in MASLIHAT arm.

^b^ Difference between arms for PEs at baseline.

^c^ Difference between PEs and NMs in MASLIHAT arm at baseline.

^d^ Difference between arms for NMs at baseline.

^e^ Difference between arms for PEs at follow‐up time points.

^f^ Difference between PEs and NMs in MASLIHAT arm at follow‐up time points.

^g^ Difference between PEs and NMs in TANSIHAT (control) arm at follow‐up time points.

**Table 3:** Intervention effects on sexual risk behaviours, unadjusted mixed effects robust Poisson regression

	Any condomless sex			Multiple sex partners			Sexual activity w/sex workers		
	*IRR*	*95% CI*	*p‐value*	*IRR*	*95% CI*	*p‐value*	*IRR*	*95% CI*	*p‐value*
Time^a^									
3 Months	0.50	0.34, 0.73	<0.001	0.28	0.17, 0.45	<0.001	0.74	0.66, 0.84	<0.001
6 Months	0.60	0.42, 0.86	0.005	0.27	0.17, 0.44	<0.001	0.78	0.68, 0.89	<0.001
9 Months	0.65	0.45, 0.95	0.026	0.26	0.15, 0.45	<0.001	0.69	0.57, 0.84	<0.001
12 Months	0.67	0.46, 0.97	0.035	0.34	0.19, 0.61	<0.001	0.75	0.61, 0.92	0.005
Arm^b^									
TANSIHAT versus MASLIHAT	1.75	1.24, 2.46	0.001	1.02	0.63, 1.64	0.951	1.06	0.74, 1.52	0.752
Participant type^c^									
Network member versus PE	1.02	0.75, 1.40	0.894	1.17	0.80, 1.70	0.43	1.69	1.26	2.25
Arm × Participant type^d^	0.39	0.25, 0.62	<0.001	−			−		
Time × Arm^e^									
3 Months	1.67	1.10, 2.53	0.018	3.04	1.96, 4.71	<0.001	1.39	1.18, 1.63	<0.001
6 Months	1.81	1.24, 2.62	0.002	3.61	2.32, 5.63	<0.001	1.45	1.21, 1.73	<0.001
9 Months	1.56	1.05, 2.32	0.026	3.78	2.31, 6.18	<0.001	1.75	1.38, 2.22	<0.001
12 Months	1.68	1.13, 2.49	0.010	3.97	2.25, 7.00	<0.001	1.70	1.31, 2.19	<0.001
Time × Participant type^f^									
3 Months	0.53	0.31, 0.93	0.027	1.17	0.83, 1.65	0.370	−		
6 Months	1.19	0.76, 1.89	0.445	1.07	0.74, 1.52	0.729			
9 Months	1.11	0.75, 1.63	0.609	1.04	0.70, 1.55	0.832			
12 Months	1.14	0.75, 1.73	0.550	0.72	0.49, 1.05	0.091			
Time × Arm × Participant type^g^									
3 Months	2.14	1.16, 3.94	0.015	−			−		
6 Months	1.22	0.72, 2.05	0.461						
9 Months	1.60	1.00, 2.54	0.049						
12 Months	1.77	1.06, 2.97	0.030						
Random intercept variances	*var*	*SE*		*var*	*SE*		*var*	*SE*	
cluster	0.18	0.065		0.89	0.288		0.37	0.143	
subject	0.24	0.094		1.37	0.322		0.92	0.213	
*N*	420			420			420		
clusters	140			140			140		
observations	2043			2043			2040		

Abbreviations: CI, confidence interval; IRR, incidence rate ratio; MASLIHAT, “Migrants’ Approached Self‐Learning Intervention in HIV/AIDS for Tajiks”; NM, network member; PE, peer educator; SE, standard error; TANSIHAT, Targeted Application of Network and Social Intervention on Health Assistance for Tajiks (comparison condition); var, variance.

^a^ Effect of time for PEs in MASLIHAT arm.

^b^ Difference between arms for PEs at baseline.

^c^ Difference between PEs and NMs in MASLIHAT arm at baseline.

^d^ Difference between arms for NMs at baseline.

^e^ Difference between arms for PEs at follow‐up time points.

^f^ Difference between PEs and NMs in MASLIHAT arm at follow‐up time points.

^g^ Difference between PEs and NMs in TANSIHAT (control) arm at follow‐up time points.

**Table 4:** Intervention effects on alcohol use, unadjusted mixed effects robust Poisson regression

	Monthly binge alcohol			Days drinking alcohol		
	*IRR*	*95% CI*	*p‐value*	*IRR*	*95% CI*	*p‐value*
Time^a^						
3 Months	0.70	0.48, 1.02	0.065	0.86	0.79, 0.94	0.001
6 Months	0.65	0.45, 0.94	0.022	0.86	0.77, 0.95	0.004
9 Months	0.63	0.43, 0.92	0.016	1.14	0.91, 1.43	0.264
12 Months	0.67	0.47, 0.95	0.026	1.19	0.98, 1.45	0.085
Arm^b^						
TANSIHAT versus MASLIHAT	2.59	0.95, 7.06	0.063	1.07	0.79, 1.44	0.665
Participant type^c^						
Network member versus PE	2.44	1.00, 5.98	0.051	1.34	1.06, 1.68	0.013
Arm × Participant type^d^	0.20	0.06, 0.68	0.010	0.89	0.67, 1.18	0.429
Time × Arm^e^						
3 Months	1.28	0.91, 1.79	0.154	1.15	1.02, 1.29	0.024
6 Months	1.33	0.95, 1.86	0.092	1.20	1.03, 1.40	0.017
9 Months	1.29	0.93, 1.80	0.125	0.89	0.68, 1.17	0.413
12 Months	1.30	0.94, 1.78	0.110	1.04	0.81, 1.33	0.763
Time × Participant type^f^						
3 Months	0.95	0.70, 1.28	0.723	1.00	0.91, 1.09	0.919
6 Months	1.00	0.75, 1.33	0.985	1.06	0.95, 1.18	0.315
9 Months	1.06	0.77, 1.44	0.746	0.91	0.75, 1.11	0.360
12 Months	1.01	0.76, 1.34	0.967	0.91	0.76, 1.09	0.310
Time × Arm × Participant type^g^						
3 Months	−			0.96	0.85, 1.09	0.524
6 Months				0.89	0.76, 1.04	0.131
9 Months				1.08	0.84, 1,38	0.569
12 Months				0.97	0.76, 1.24	0.804
Random intercept variances	*var*	*SE*		*var*	*SE*	
cluster	0	0		0.16	0.08	
subject	7.12	0.82		0.13	0.040	
*N*	420			420		
clusters	140			140		
observations	2036			2038		

Abbreviations: CI, confidence interval; IRR, incidence rate ratio; MASLIHAT, “Migrants’ Approached Self‐Learning Intervention in HIV/AIDS for Tajiks”; NM, network member; PE, peer educator; SE, standard error; TANSIHAT, Targeted Application of Network and Social Intervention on Health Assistance for Tajiks (comparison condition); var, variance.

^a^ Effect of time for PEs in MASLIHAT arm.

^b^ Difference between arms for PEs at baseline.

^c^ Difference between PEs and NMs in MASLIHAT arm at baseline.

^d^ Difference between arms for NMs at baseline.

^e^ Difference between arms for PEs at follow‐up time points.

^f^ Difference between PEs and NMs in MASLIHAT arm at follow‐up time points.

^g^ Difference between PEs and NMs in TANSIHAT (control) arm at follow‐up time points.

The unadjusted results for injection risk behaviour are shown in Table 2. The time × condition interaction for RSS was significant for both MASLIHAT PEs (Chi2[4] = 105.91, *p* < 0.0001) and NMs (Chi2[4] = 256.12, *p* < 0.0001). Significant declines in RSS were sustained over 12 months of follow‐up (see Figure 2). Similar results were seen for ancillary equipment sharing for PEs (Chi2[4] = 118.89, *p* < 0.0001) and NMs (Chi2[4] = 134.74, *p* < 0.0001). Covariate adjustment had little effect on estimates and did not alter the conclusions.

The results of the unadjusted Poisson models for sexual risk behaviour are shown in Table 3. There were initially significant declines in the prevalence of condomless sex among MASLIHAT versus TANSIHAT PEs (Chi2[4] = 14.64, *p* = 0.0055) and NMs (Chi2[4] = 53.39, *p*<0.0001). At 12‐month follow‐up, the unadjusted prevalence of condomless sex was only modestly lower than baseline (PE: dy/dx = −0.14, z = −2.21, *p* = 0.027; NM: dy/dx = −0.10, z = −1.94, *p* = 0.053). When adjusted, however, for age and level of education (see File S1), the decline in prevalence of condomless sex appeared sustained (see Figure 3). The prevalence of multiple female sex partners declined significantly following the intervention and in contrast to the control condition for PEs (Chi2[4] = 18.10, *p* = 0.0012) and NMs (Chi2[4] = 28.01, *p* < 0.0001) and was sustained over 12 months (see Figure 4). The prevalence of sex with sex workers also declined for MASLIHAT PEs (Chi2[4] = 17.77, *p* = 0.0014) and NMs (Chi2[4] = 24.23, *p* = 0.0001) and the effect was sustained over 12 months. Covariate adjustment had little effect on estimates of multiple partners or commercial sex and did not alter the conclusions.

The results of the unadjusted Poisson models for alcohol use measures are shown in Table 4. The intervention had no effect on monthly binge drinking (Chi2[8] = 14.48, *p* = 0.07). Frequency of alcohol use (days drinking past 30 days) initially decreased among MASLIHAT versus control PEs (Chi2[4] = 14.56, *p* = 0.0057) with less effect among NMs (Chi2[4] = 10.98, *p* = 0.0268). At 9‐month follow‐up, drinking days increased significantly with levels rising above baseline at 12 months (see Figure 5). Covariate adjustment had little effect on estimates and did not alter the conclusions.

## 4 DISCUSSION

The MASLIHAT intervention for HIV prevention is a network‐based peer education intervention, tailored for male Tajik migrants who inject drugs while working in Russia. In this cluster‐randomized controlled trial, we found significant reductions in self‐reported injection and sexual risk behaviours associated with intervention participation when compared with a time‐matched control intervention with referral in both conditions to HIV testing and counselling. Changes in behaviour that persisted for 12 months were reported by both PEs who attended the MASLIHAT intervention sessions and their NMs to whom they relayed the information they had learned. Reductions in injection risk behaviour among both PEs and NMs were quite dramatic, while changes in sexual risk behaviour were less pronounced. Peer network intervention studies in the United States similarly found stronger results for injection than for sexual risk behaviour [25, 26, 29].

The observed differences in positive change between injection versus sexual behaviour may be due in part to the situations and settings in which they occur. Injection drug use is a shared behaviour, while sexual encounters typically occur in private. It is not surprising that network norms of risk reduction are more likely to influence behaviours conducted in an observable space. It is also possible that condom use is not sustained once a monogamous sexual relationship is established [30]. A more nuanced definition of sexual risk may better capture behavioural changes.

Although the intervention aimed to reduce heavy alcohol use associated with HIV risk behaviour through disinhibition, we saw only transitory reductions in the frequency of drinking and no effect on binge alcohol use. While there was a slight decline in binge drinking in both groups, the decline was only nominally greater in the MASLIHAT group (20 percentage points vs. 10 percentage points in TANSIHAT). Additional analyses are warranted to explore if the observed changes in participant alcohol use are concentrated among participants who have problem drinking (e.g. high AUDIT scores at baseline). Since the TANSIHAT intervention also teaches about healthy lifestyles including safer alcohol use, there may not be a detectable difference between groups. Nevertheless, over half of all participants continued to report binge drinking at least once a month. This suggests that a different approach may be needed to address hazardous drinking. The observed increase in drinking frequency at 9 months roughly coincides with the Russian invasion of Ukraine and subsequent sanctions against Russia. These sanctions affected Tajik migrants both economically and socially. The added stress that Tajik migrants likely experienced during this turbulent period may have increased their level and frequency of alcohol consumption.

Community‐based programmes employing peer outreach workers (peer‐led outreach) have proved successful in changing behaviour and disseminating prevention information to PWID in other countries [31, 32, 33]. Meanwhile, peer network interventions have been tested with PWID in the United States, Vietnam, Thailand and in St. Petersburg, Russia, but not with temporary labour migrants [28, 29, 34, 35, 36, 37]. The MASLIHAT intervention is distinctive in being culturally adapted for Tajik male migrant workers at risk for HIV through injecting drugs and in addressing the challenges they face due to social marginalization and economic disadvantage. The MASLIHAT intervention is potentially generalizable to migrant populations of PWID in other Russian cities and other countries if adapted for cultural context and the local situation just as we adapted SHIELD to fit the social environment and life circumstances of Tajik migrants in Moscow [9].

### 4.1 Limitations

The intervention was tested with male migrants only, and did not include any assessment of same‐sex behaviour. Due to the strong social stigma among Tajik migrants towards same‐sex behaviour, its prevalence and HIV risks are difficult to assess reliably. Additional work that is population‐appropriate and culturally acceptable is required to evaluate MASLIHAT's impact on Tajik migrant male‐to‐male sexual risk behaviour. The primary outcomes in this study relied on self‐reported behaviour. Self‐reports cannot be verified and can be subject to the demand characteristics of the intervention or to social desirability bias. The results of HIV testing were not individually identifiable, and self‐disclosure during follow‐up was inconsistent. Consequently, HIV status could not be included in the regression models predicting risk behaviour.

## 5 CONCLUSIONS

The MASLIHAT peer‐education intervention has proved highly effective in reducing HIV risk through injection drug use and moderately effective in reducing sexual risk among male Tajik intervention participants and their NMs. Given its demonstrated success, it is likely that the MASLIHAT intervention model if culturally adapted holds the potential for increasing HIV prevention among other central Asia migrant populations known to inject drugs in Russia and in other global destination countries where populations of migrant PWID are at high risk for acquiring HIV.

## COMPETING INTERESTS

The authors have no competing interests to declare.

## AUTHORS’ CONTRIBUTIONS

JAL, MEM‐A and MB contributed to the study conception and design. Material preparation and data collection were performed by JJ and CML. Data analysis was conducted by MEM‐A and CML. The first draft of the manuscript was written by MEM‐A and all authors commented on previous versions of the manuscript. All authors read and approved the final manuscript.

## FUNDING

This research was supported by a grant from the National Institute on Drug Abuse of the National Institutes of Health (USA) under Award Number R01DA050464 and by a grant from the National Center for Advancing Translational Science, NIH, through grant UL1TR002003.

## DISCLAIMER

The content is solely the responsibility of the authors and does not necessarily represent the official views of the National Institutes of Health.

## Supporting information


**File S1**: Additional tables

## Data Availability

The datasets supporting the findings of this study are available in the Open Science Framework repository. Project: MASLIHAT Randomized Controlled Trial [DOI 10.17605/OSF.IO/7G3YH. Data link: https://osf.io/krjqs/?view_only=7c93ea8cf8384dce91b2b531000e63e3].
