# Supplementary material for: Changes in risk behaviour following a network peer education intervention for HIV prevention among male Tajik migrants who inject drugs in Moscow: a cluster‐randomized controlled trial
Source: J Int AIDS Soc. 2024 Jul 19;27(Suppl 3):e26310. doi: 10.1002/jia2.26310 (PMC11258451; doi:10.1002/jia2.26310)
Supplement: Supplementary file 1 — File S1: Additional tables [file JIA2-27-e26310-s001.docx]

**Changes in Risk Behavior Following a Network Peer Education Intervention for HIV Prevention Among Male Tajik Migrants Who Inject Drugs in Moscow: A Cluster-randomized Controlled Trial**

**Additional Tables**

Covariate-adjusted mixed effects Poisson regression models

| **Table S1. Intervention effects on injection risk behaviors: covariate adjusted mixed effects robust Poisson regression** | | | | | | |
| --- | --- | --- | --- | --- | --- | --- |
|  | **Syringe Sharing** | | | **Equipment Sharing** | | |
|  | *IRR* | *95% CI* | *p-value* | *IRR* | *95% CI* | *p-value* |
| Time ^†^ |  |  |  |  |  |  |
| 3 Months | 0.13 | 0.06, 0.28 | < 0.001 | 0.11 | 0.07, 0.17 | < 0.001 |
| 6 Months | 0.07 | 0.02, 0.20 | < 0.001 | 0.11 | 0.07, 0.18 | < 0.001 |
| 9 Months | 0.14 | 0.06, 0.29 | < 0.001 | 0.11 | 0.06, 0.19 | < 0.001 |
| 12 Months | 0.31 | 0.19, 0.50 | < 0.001 | 0.12 | 0.07, 0.19 | < 0.001 |
| Arm ^‡^ |  |  |  |  |  |  |
| Intervention vs Control | 1 | 0.79, 1.27 | 0.981 | 0.99 | 0.82, 1.19 | 0.905 |
| Participant Type § |  |  |  |  |  |  |
| Network member vs PE | 1.19 | 1.01, 1.41 | 0.038 | 1.08 | 0.97, 1.21 | 0.165 |
| Arm x Participant type ¶ | 1.03 | 0.81, 1.30 | 0.835 |  |  |  |
| Time x Arm †† |  |  |  |  |  |  |
| 3 Months | 8.16 | 3.77, 17.62 | < 0.001 | 10.56 | 6.70, 16.65 | < 0.001 |
| 6 Months | 17.94 | 5.80, 55.47 | < 0.001 | 10.74 | 6.32, 18.25 | <0.001 |
| 9 Months | 8.64 | 3.98, 18.76 | < 0.001 | 10.89 | 6.18, 19.19 | <0.001 |
| 12 Months | 3.72 | 2.28, 6.06 | < 0.001 | 10.64 | 6.44, 17.59 | <0.001 |
| Time x Participant type ‡‡ |  |  |  |  |  |  |
| 3 Months | 0.70 | 0.31, 1.54 | 0.374 | - |  |  |
| 6 Months | 1.53 | 0.47, 5.04 | 0.482 |  |  |  |
| 9 Months | 1.05 | 0.54, 2.07 | 0.881 |  |  |  |
| 12 Months | 0.73 | 0.50, 1.07 | 0.109 |  |  |  |
| Time x Arm x Participant type §§ |  |  |  |  |  |  |
| 3 Months | 1.40 | 0.63, 3.10 | 0.414 | - |  |  |
| 6 Months | 0.59 | 0.18, 1.94 | 0.383 |  |  |  |
| 9 Months | 0.84 | 0.42, 1.67 | 0.616 |  |  |  |
| 12 Months | 1.27 | 0.85, 1.90 | 0.247 |  |  |  |
| Time in Moscow (years) |  |  |  |  |  |  |
| >1 to 2 | 1.08 | 0.87, 1.34 | 0.475 |  |  |  |
| >2 to 3 | 1.19 | 0.96, 1.47 | 0.115 |  |  |  |
| >3 | 1.19 | 0.96, 1.47 | 0.112 |  |  |  |
| *vs. 1 year or less* |  |  |  |  |  |  |
| Trips |  |  |  |  |  |  |
| Two |  |  |  | 0.98 | 0.83, 1.17 | 0.863 |
| Three |  |  |  | 0.97 | 0.80, 1.16 | 0.713 |
| Four or more |  |  |  | 0.95 | 0.79, 1.15 | 0.598 |
| *vs. One* |  |  |  |  |  |  |
| Education |  |  |  |  |  |  |
| Secondary |  |  |  | 1.09 | 0.82, 1.44 | 0.547 |
| Higher Education |  |  |  | 1.09 | 0.83, 1.44 | 0.528 |
| *vs. Primary* |  |  |  |  |  |  |
| Random Intecept Variances | *var* | *SE* |  | *var* | *SE* |  |
| cluster | 0 | 0 |  | 0 | 0 |  |
| subject | 0 | 0 |  | 0 | 0 |  |
|  |  |  |  |  |  |  |
| N | 416 |  |  | 420 |  |  |
| clusters | 140 |  |  | 140 |  |  |
| obs | 2021 |  |  | 1973 |  |  |
| † effect of time for PEs in MASLIHAT arm; ‡ difference between arms for PEs at baseline; § difference between PEs and NWs in MASLIHAT arm at baseline; ¶ difference between arms for NMs at baseline; †† difference between arms for PEs at follow-up time points; ‡‡ difference between PEs and NMs in MASLIHAT arm at follow-up time points; §§ difference between PEs and NMs in TANSIHAT (control) arm at follow-up time points  PE: peer educator; NM: network member | | | | | | |

| **Table S2. Intervention effects on sexual risk behaviors: covariate adjusted mixed effects robust Poisson regression** | | | | | | | | | |
| --- | --- | --- | --- | --- | --- | --- | --- | --- | --- |
|  | **Any Condomless Sex** | | | **Multiple Sex Partners** | | | **Sexual Activity w/ Sex Workerss** | | |
|  | *IRR* | *95% CI* | *p-value* | *IRR* | *95% CI* | *p-value* | *IRR* | *95% CI* | *p-value* |
| Time † |  |  |  |  |  |  |  |  |  |
| 3 Months | 0.43 | 0.29, 0.65 | < 0.001 | 0.28 | 0.17, 0.45 | < 0.001 | 0.74 | 0.66, 0.84 | < 0.001 |
| 6 Months | 0.47 | 0.32, 0.69 | < 0.001 | 0.27 | 0.17, 0.43 | < 0.001 | 0.78 | 0.68, 0.89 | < 0.001 |
| 9 Months | 0.38 | 0.24, 0.60 | < 0.001 | 0.26 | 0.16, 0.45 | < 0.001 | 0.69 | 0.57, 0.84 | < 0.001 |
| 12 Months | 0.45 | 0.28, 0.72 | 0.001 | 0.34 | 0.19, 0.61 | < 0.001 | 0.75 | 0.61, 0.92 | 0.005 |
| Arm ‡ |  |  |  |  |  |  |  |  |  |
| Intervention vs Control | 2.12 | 1.44, 3.12 | < 0.001 | 1.13 | 0.81, 1.57 | 0.475 | 1.10 | 0.83, 1.46 | 0.496 |
| Participant Type § |  |  |  |  |  |  |  |  |  |
| Network member vs PE | 0.93 | 0.65, 1.33 | 0.677 | 1.02 | 0.69, 1.50 | 0.932 | 1.54 | 1.15, 2.07 | 0.004 |
| Arm x Participant type ¶ | 0.31 | 0.18, 0.53 | < 0.001 |  |  |  |  |  |  |
| Time x Arm †† |  |  |  |  |  |  |  |  |  |
| 3 Months | 1.92 | 1.23, 3.00 | 0.004 | 3.04 | 1.96, 4.72 | < 0.001 | 1.39 | 1.18, 1.63 | < 0.001 |
| 6 Months | 2.19 | 1.47, 3.23 | < 0.001 | 3.61 | 2.32, 5.63 | < 0.001 | 1.45 | 1.21, 1.73 | < 0.001 |
| 9 Months | 2.54 | 1.57, 4.10 | < 0.001 | 3.77 | 2.30, 6.16 | < 0.001 | 1.76 | 1.39, 2.24 | < 0.001 |
| 12 Months | 2.36 | 1.47, 3.79 | < 0.001 | 3.98 | 2.25, 7.05 | < 0.001 | 1.71 | 1.33, 2.22 | < 0.001 |
| Time x Participant type ‡‡ |  |  |  |  |  |  |  |  |  |
| 3 Months | 0.59 | 0.32, 1.09 | 0.089 | 1.18 | 0.84, 1.65 | 0.346 | - |  |  |
| 6 Months | 0.43 | 0.23, 0.81 | 0.009 | 1.07 | 0.75, 1.53 | 0.697 |  |  |  |
| 9 Months | 0.69 | 0.37, 1.30 | 0.252 | 1.05 | 0.71, 1.55 | 0.808 |  |  |  |
| 12 Months | 0.61 | 0.32, 1.14 | 0.119 | 0.72 | 0.49,1.05 | 0.091 |  |  |  |
| Time x Arm x Participant type §§ |  |  |  |  |  |  |  |  |  |
| 3 Months | 2.04 | 1.06, 3.95 | 0.034 | - |  |  | - |  |  |
| 6 Months | 2.59 | 1.33, 5.04 | 0.005 |  |  |  |  |  |  |
| 9 Months | 1.79 | 0.90, 3.57 | 0.096 |  |  |  |  |  |  |
| 12 Months | 2.04 | 1.03, 4.05 | 0.041 |  |  |  |  |  |  |
| Time in Moscow (years) |  |  |  |  |  |  |  |  |  |
| >1 to 2 |  |  |  | 0.89 | 0.51, 1.54 | 0.667 |  |  |  |
| >2 to 3 |  |  |  | 0.95 | 0.54, 1.67 | 0.87 |  |  |  |
| >3 |  |  |  | 0.65 | 0.35, 1.24 | 0.192 |  |  |  |
| *vs. 1 year or less* |  |  |  |  |  |  |  |  |  |
| Trips |  |  |  |  |  |  |  |  |  |
| 2 |  |  |  | 1.30 | 0.77, 2.21 | 0.327 | 1.08 | 0.75, 1.57 | 0.669 |
| 3 |  |  |  | 0.97 | 0.55, 1.72 | 0.928 | 1.04 | 0.71, 1.53 | 0.840 |
| 4 or more |  |  |  | 0.64 | 0.32, 1.28 | 0.208 | 0.67 | 0.39, 1.14 | 0.139 |
| *vs. 1* |  |  |  |  |  |  |  |  |  |
| Age | 0.97 | 0.95, 1.00 | 0.052 | 0.87 | 0.84, 0.91 | < 0.001 | 0.93 | 0.91, 0.96 | < 0.001 |
| Education |  |  |  |  |  |  |  |  |  |
| Secondary | 3.62 | 1.08, 12.08 | 0.037 | 4.73 | 1.64, 13.67 | 0.004 |  |  |  |
| Higher Education | 1.48 | 0.44, 5.03 | 0.529 | 4.58 | 1.47, 14.33 | 0.009 |  |  |  |
| *vs. Primary* |  |  |  |  |  |  |  |  |  |
| Random Intecept Variances | *var* | *SE* |  | *var* | *SE* |  | *var* | *SE* |  |
| cluster | 0 | 0 |  | 0.02 | 0.11 |  | 0.02 | 0.09 |  |
| subject | 1.04 | 0.19 |  | 1.29 | 0.28 |  | 0.95 | 0.21 |  |
|  |  |  |  |  |  |  |  |  |  |
| N | 420 |  |  | 416 |  |  | 420 |  |  |
| clusters | 140 |  |  | 140 |  |  | 140 |  |  |
| obs | 2043 |  |  | 2025 |  |  | 2040 |  |  |
| † effect of time for PEs in MASLIHAT arm; ‡ difference between arms for PEs at baseline; § difference between PEs and NWs in MASLIHAT arm at baseline; ¶ difference between arms for NMs at baseline; †† difference between arms for PEs at follow-up time points; ‡‡ difference between PEs and NMs in MASLIHAT arm at follow-up time points; §§ difference between PEs and NMs in TANSIHAT (control) arm at follow-up time points  PE: peer educator; NM: network member | | | | | | | | | |

| **Table S3. Intervention effects on alcohol use: covariate adjusted mixed effects robust Poisson regression** | | | | | | |
| --- | --- | --- | --- | --- | --- | --- |
|  | **Binge Alcohol Consumption (Monthly)** | | | **Days Drinking Alcohol** | | |
|  | *IRR* | *95% CI* | *p-value* | *IRR* | *95% CI* | *p-value* |
| Time † |  |  |  |  |  |  |
| 3 Months | 0.71 | 0.48, 1.04 | 0.075 | 0.86 | 0.79, 0.94 | < 0.001 |
| 6 Months | 0.66 | 0.46, 0.95 | 0.026 | 0.86 | 0.77, 0.95 | 0.003 |
| 9 Months | 0.64 | 0.44, 0.93 | 0.019 | 1.14 | 0.91, 1.44 | 0.262 |
| 12 Months | 0.68 | 0.48, 0.97 | 0.032 | 1.19 | 0.97, 1.45 | 0.091 |
| Arm ‡ |  |  |  |  |  |  |
| Intervention vs Control | 2.39 | 0.85, 6.68 | 0.097 | 1.07 | 0.79, 1.44 | 0.657 |
| Participant Type § |  |  |  |  |  |  |
| Network member vs PE | 2.22 | 0.90, 5.46 | 0.083 | 1.34 | 1.06, 1.69 | 0.015 |
| Arm x Participant type ¶ | 0.24 | 0.07, 0.84 | 0.026 | 0.89 | 0.67, 1.19 | 0.429 |
| Time x Arm †† |  |  |  |  |  |  |
| 3 Months | 1.25 | 0.90, 1.75 | 0.188 | 1.15 | 1.02, 1.30 | 0.024 |
| 6 Months | 1.31 | 0.94, 1.82 | 0.114 | 1.20 | 1.03, 1.40 | 0.017 |
| 9 Months | 1.27 | 0.91, 1.76 | 0.155 | 0.89 | 0.68, 1.17 | 0.413 |
| 12 Months | 1.27 | 0.93, 1.73 | 0.139 | 1.04 | 0.81, 1.34 | 0.763 |
| Time x Participant type ‡‡ |  |  |  |  |  |  |
| 3 Months | 0.96 | 0.71, 1.29 | 0.776 | 1 | 0.91, 1.09 | 0.937 |
| 6 Months | 1.01 | 0.76, 1.34 | 0.956 | 1.06 | 0.95, 1.18 | 0.306 |
| 9 Months | 1.06 | 0.78, 1.45 | 0.693 | 0.91 | 0.75, 1.11 | 0.356 |
| 12 Months | 1.02 | 0.77, 1.34 | 0.917 | 0.91 | 0.76, 1.09 | 0.317 |
| Time x Arm x Participant type §§ |  |  |  |  |  |  |
| 3 Months |  |  |  | 0.96 | 0.84, 1.09 | 0.508 |
| 6 Months |  |  |  | 0.89 | 0.76, 1.04 | 0.127 |
| 9 Months |  |  |  | 1.08 | 0.84, 1.39 | 0.563 |
| 12 Months |  |  |  | 0.97 | 0.75, 1.24 | 0.805 |
| Time in Moscow (years) |  |  |  |  |  |  |
| >1 to 2 | 5.60 | 0.36, 86.55 | 0.217 | 1.09 | 0.91, 1.32 | 0.355 |
| >2 to 3 | 10.79 | 0.71, 163.71 | 0.087 | 1.18 | 0.98, 1.43 | 0.085 |
| >3 | 15.70 | 1.07, 231.28 | 0.045 | 1.29 | 1.07, 1.55 | 0.006 |
| *vs. 1 year or less* |  |  |  |  |  |  |
| Education |  |  |  |  |  |  |
| Secondary |  |  |  | 0.88 | 0.74, 1.05 | 0.146 |
| Higher Education |  |  |  | 0.94 | 0.80, 1.11 | 0.476 |
| *vs. Primary* |  |  |  |  |  |  |
| Random Intecept Variances | *var* | *SE* |  | *var* | *SE* |  |
| cluster | 0 | 0 |  | 0.14 | 0.070 |  |
| subject | 6.49 | 0.76 |  | 0.13 | 0.040 |  |
|  |  |  |  |  |  |  |
| N | 416 |  |  | 416 |  |  |
| clusters | 140 |  |  | 140 |  |  |
| obs | 2018 |  |  | 2020 |  |  |
| † effect of time for PEs in MASLIHAT arm; ‡ difference between arms for PEs at baseline; § difference between PEs and NWs in MASLIHAT arm at baseline; ¶ difference between arms for NMs at baseline; †† difference between arms for PEs at follow-up time points; ‡‡ difference between PEs and NMs in MASLIHAT arm at follow-up time points; §§ difference between PEs and NMs in TANSIHAT (control) arm at follow-up time points  PE: peer educator; NM: network member | | | | | | |
